# *Granopupa* in Iran, monophyly, and the fossil Granariinae (Gastropoda, Pulmonata, Chondrinidae)

**DOI:** 10.3897/zookeys.592.7907

**Published:** 2016-05-25

**Authors:** Edmund Gittenberger, Bas Kokshoorn, Ulrich Bößneck, Bastian T. Reijnen, Dirk S. J. Groenenberg

**Affiliations:** 1 Naturalis Biodiversity Center, P.O. Box 9517, NL-2300RA Leiden, The Netherlands; 2 Natural History Museum of Erfurt, Grosse Arche 14, D-99084 Erfurt, Germany

**Keywords:** Granopupa persica, Graniberia, taxonomy, anatomy, COI, 16S

## Abstract

Indisputable Chondrinidae, Granariinae species, characterized by shell shape and apertural dentition, are known from Eocene deposits to the Recent. The generic classification of the extant species is based on conchological, anatomical and molecular data that are available now for most of the known species, including *‘Granaria’ persica* as a representative of the once problematic group of so-called eastern *Granaria* species. According to molecular and anatomical characters, these eastern species have to be classified with *Granopupa granum* in *Granopupa*. *Graniberia* **gen. n.** is introduced for *Granaria braunii* on the basis of molecular and conchological data. For the pre-Pleistocene species, two generic names are equally well available now, viz. *Granopupa* and *Granaria*. Shell characters only do not enable a decision here. For the sake of nomenclatorial stability we propose to use *Granaria* for these species. Because both molecular and anatomical data most likely will never be known for the fossils, it will remain unclear whether the combined extant and extinct *Granaria* species form a monophyletic group.

## Introduction

The genus-group taxa of the Chondrinidae Steenberg, 1925, are currently characterized by conchological, anatomical, and molecular characters (Gittenberger 1973, Kokshoorn and Gittenberger 2008, 2010). These data were not available for all the species, however, so that not all could be classified accordingly.

The extant *Granopupa granum* (Draparnaud, 1801), and *Granaria* Held, 1837, species, and all the fossil chondrinids known from Eocene to Pliocene, have similar shell shapes and, what is more distinctive, the same characteristic arrangement of the apertural teeth, i.e. the palatalis inferior is more prominently developed than the other palatals. The extant so-called *Granaria* species show a disjunct distribution, with a western group of four species occurring in Europe and an eastern group of three species in the Arabian peninsula and Iran. The western species, viz. *Granaria frumentum*, *Granaria variabilis*, *Granaria stabilei* and *Granaria braunii*, are relatively well-known, whereas the eastern group, viz. *Granopupa lapidaria*, *Granaria persica* and *Granopupa arabica*, was known from shells only.

The shells of *Granopupa granum*, measuring 3.1–6.0 × 1.4–1.8 mm, are smaller than those of the European *Granaria* species, measuring 5.6–9.3 × 2.1–2.6 mm in the smallest, i.e. *Granaria stabilei*, and 6.7–19.0 × 2.6–4.5 in the largest species, i.e. *Granaria variabilis* (see Gittenberger 1973). The generic classification of the species from the Middle East, with shells measuring 4.9–5.5 × 2.1–2.2 mm (see Gittenberger 1973), was questionable. They could be either relatively small *Granaria*, or large *Granopupa*, because an obvious gap in sizes does not exist. The use of the generic name *Granaria* for the extant species occurring in the disjunct eastern part of the alleged range of that genus, was based on tradition and on the lack of a clearly preferential alternative.

For the chondrinids from before the Pleistocene (Höltke and Rasser 2013) neither molecular nor anatomical data are likely to ever be known, so that their classification has to be based on shell morphology only.

Recently, one of us (U. B.) collected together with empty shells a live specimen of *‘Granaria’ persica*. As a consequence, both anatomical and molecular data are available for that species now. Therefore, a revised classification of this species is proposed in this article. Additionally, the generic classification of the remaining, so-called *Granaria* species is dealt with.

## Material and methods

**Figure 1.**
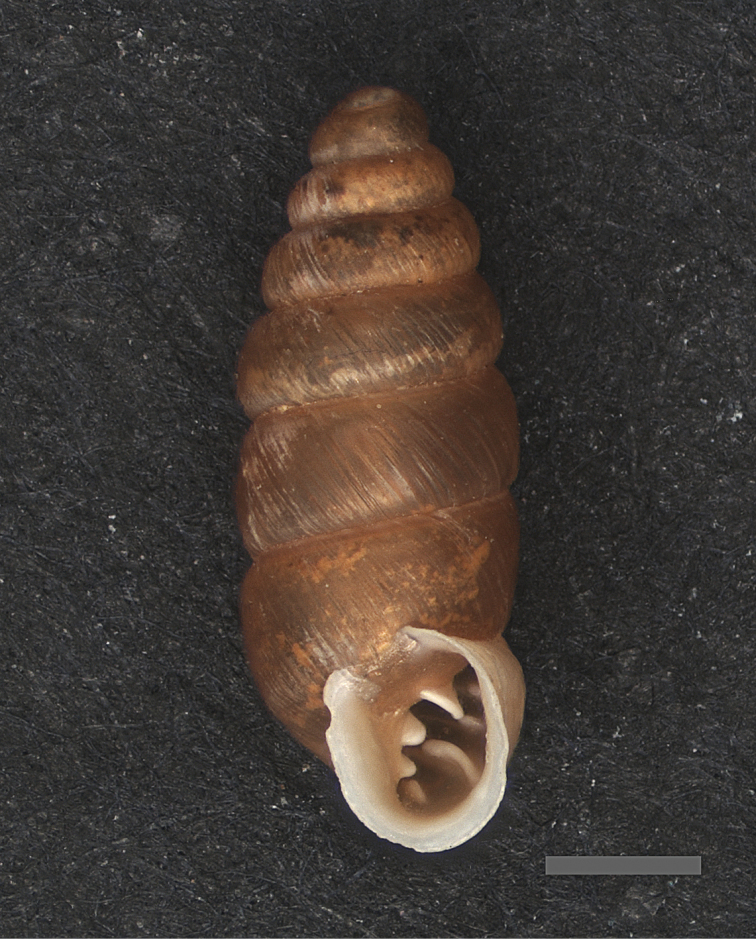
*Granopupa persica* (Gittenberger, 1973). Iran, province of Hormozgan, Kuh-e Bakhun, large valley, 980 m alt., 27°55'11"N 56°41'24"E, washed ashore; U. & K. Bößneck & A. Saboori leg. Shell height 5.2 mm (RMNH 336351). Scale bar 1 mm. Photograph by DSJG.

A single live animal of *‘Granaria’ persica* was collected by Ulrich Bößneck in Iran, province of Hormozgan, Banooband, Genu Mtn, at 740-1000 m altitude (Fig. 1). The specimen was transferred into ethanol 70%. During dissection, the proximal part of the genital tract was isolated and coloured with organic cochineal dye, subsequently hardened in ethanol 97%, then cleared in Euparal essence and eventually fixed in Euparal as a genital slide. Serial sections were not made, so that the structure of the lumen can only be described by studying the transparent parts of the genital slide with a regular microscope. The buccal mass was partly dissolved in KOH to isolate the radula, which was cleaned, mounted on a stub and photographed with a SEM.

The material that is used here is housed in Naturalis Biodiversity Center, Leiden, The Netherlands.

DNA was extracted with a DNeasy blood & tissue kit (Qiagen). *COI* and *16S* were amplified using the procedure described in Kokshoorn and Gittenberger (2008). Products were sequenced in both directions (using the same primers) at BaseClear (The Netherlands) and edited in SEQUENCHER 5 (Genecodes Corp.). Sequences for *‘Granaria’ persica* have been deposited in GenBank (accession numbers KT948999 and KT949000 for *COI* and *16S*, respectively). Datamatrices with relevant reference sequences from GenBank were constructed in Geneious PRO 7.0.6. Because no *COI* sequence is available for *Granaria variabilis* it was coded as missing data. Both datasets were aligned with MAFFT v.7.017 (Katoh and Standley 2013) using default settings. Conserved regions of the *16S* alignment were selected with GBLOCKS v. 0.91b (Castresana 2000). PARTITIONFINDER (Lanfear et al. 2012) was used to check for the best partitioning scheme (*COI* codon positions and *16S* were considered as potential partitions) and substitution models. The translated amino acid sequene of *COI* was added as a fifth partition for a Bayesian phylogeny reconstruction. None of the suggested partitions could be combined and GTR+G, GTR+G, HKY+I+G, GTR+I+G and aa mixed were specified for *COI* codon position 1, 2, 3, *16S* and the *COI* amino acid partition, respectively. A phylogenetic analysis was carried out with MRBAYES (Ronquist and Huelsenbeck 2003) v.3.2.3 (10 M generations, 2 runs, 4 chains) hosted on the CIPRES science gateway (Miller et al. 2010). Trees were sampled every 1000 generations, the first 2500 trees were discarded as burnin (relburnin = yes, burninfrac = 0.25). To compare and further explore the *COI* and *16S* datasets, both were analysed separately as well (see Supplementary information). Except for the omission of *Granaria variabilis* (for which no *COI* data are available) the MrBayes analysis (partitioning and selected models) for *COI* was identical to that for the concatenated dataset. For *16S* the complete sequences (no characters omitted) were utilized. The selected model again was GTR+I+G.

Abbreviation: pp. = posterior probability.

## Results

**Figure 2.**
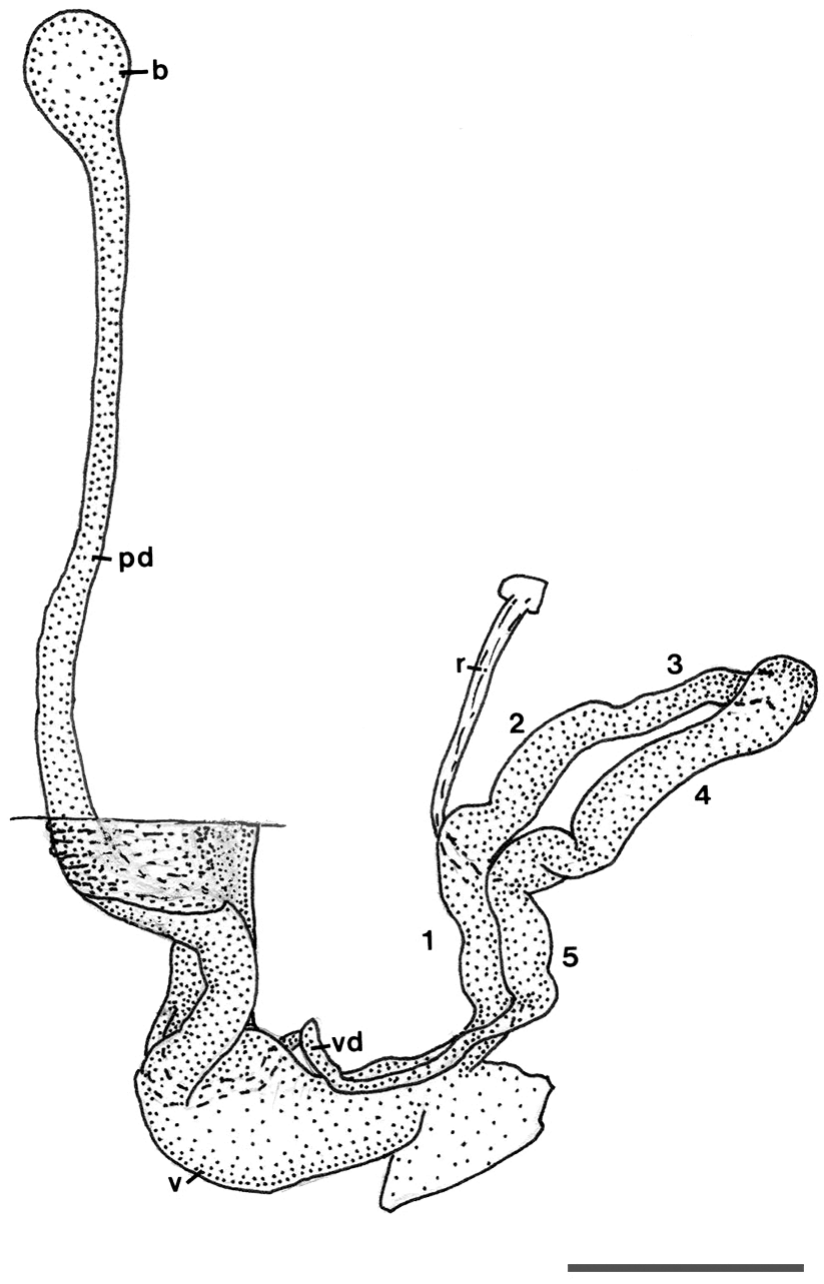
*Granopupa persica* (Gittenberger, 1973). Iran, province of Hormozgan, Genu Mt., Banooband, 740–1000 m alt., 27°22'01"N 56°09'45"E, dry rocky limestone slope with little vegetation; U. Bößneck leg. Genital tract. Abbreviations: b, bursa copulatrix; pd, pendunculus; r, retractor muscle; v, vagina; vd, vas deferens. The figures **1–5** refer to the segments of the male loop that are mentioned in the text. Scale bar 1 mm.

In *‘Granaria’ persica*, as in the other chondrinid species, the male part of the genital tract forms a loop because the proximal part of the vas deferens is fixed to the genital atrium (Gittenberger 1973). A prominent flagellum, as is present in both the *Granaria* and the *Solatopupa* species, is lacking. The male loop can be subdivided in five parts, which differ in the structure of the lumen and slightly in width; the transitional sites are more or less clearly distinguishable by irregularities in width or curvature of the duct. The segments are described from proximal (starting from the body wall) to distal. The most proximal segment of the loop, i.e. the penis (Fig. 2: 1), has a muscular wall with very fine, transverse striae and a short ridge in the distal third of the lumen. The adjoining epiphallic part (Fig. 2: 2) has a thin wall and a regular transverse structure, maybe with small papillae in the lumen. It is followed by the narrowest part of the loop (Fig. 2: 3), without any regular, luminal structure. The next part (Fig. 2: 4) is clearly broader again; the lumen has relatively large papillae, which gradually pass into a more transverse arrangement. The most distal part of the loop (Fig. 2: 5) has a thick wall with very fine transverse and longitudinal striae, and could be considered a part of the vas deferens; the longitudinal striae can be followed over some distance also more proximally.

**Figure 3.**
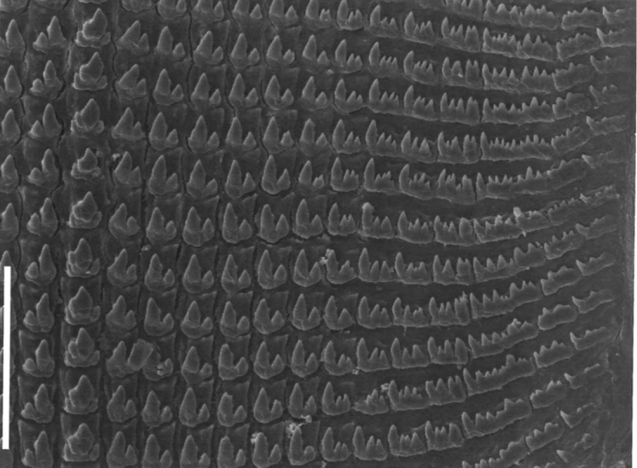
*Granopupa persica* (Gittenberger, 1973). Iran, province of Hormozgan, Genu Mt., Banooband, 740–1000 m alt., 27°22'01"N 56°09'45"E, dry rocky limestone slope with little vegetation; U. Bößneck leg. Half row of radula teeth; scale bar 50 µm. SEM photograph by L.P. van Ofwegen.

The radula of *‘Granaria’ persica* has rows of teeth with a tricuspid central tooth, accompanied by adjoining bicuspid teeth, and teeth with more cusps, towards the margin of the radular ribbon. In the specimen that could be studied, the central tooth shows some individual irregularities. It is accompanied by 6 bicuspid teeth; from tooth 7 on, the side cusp is split into two, and more marginally in more, irregular, smaller cusps (Fig. 3).

**Figure 4.**
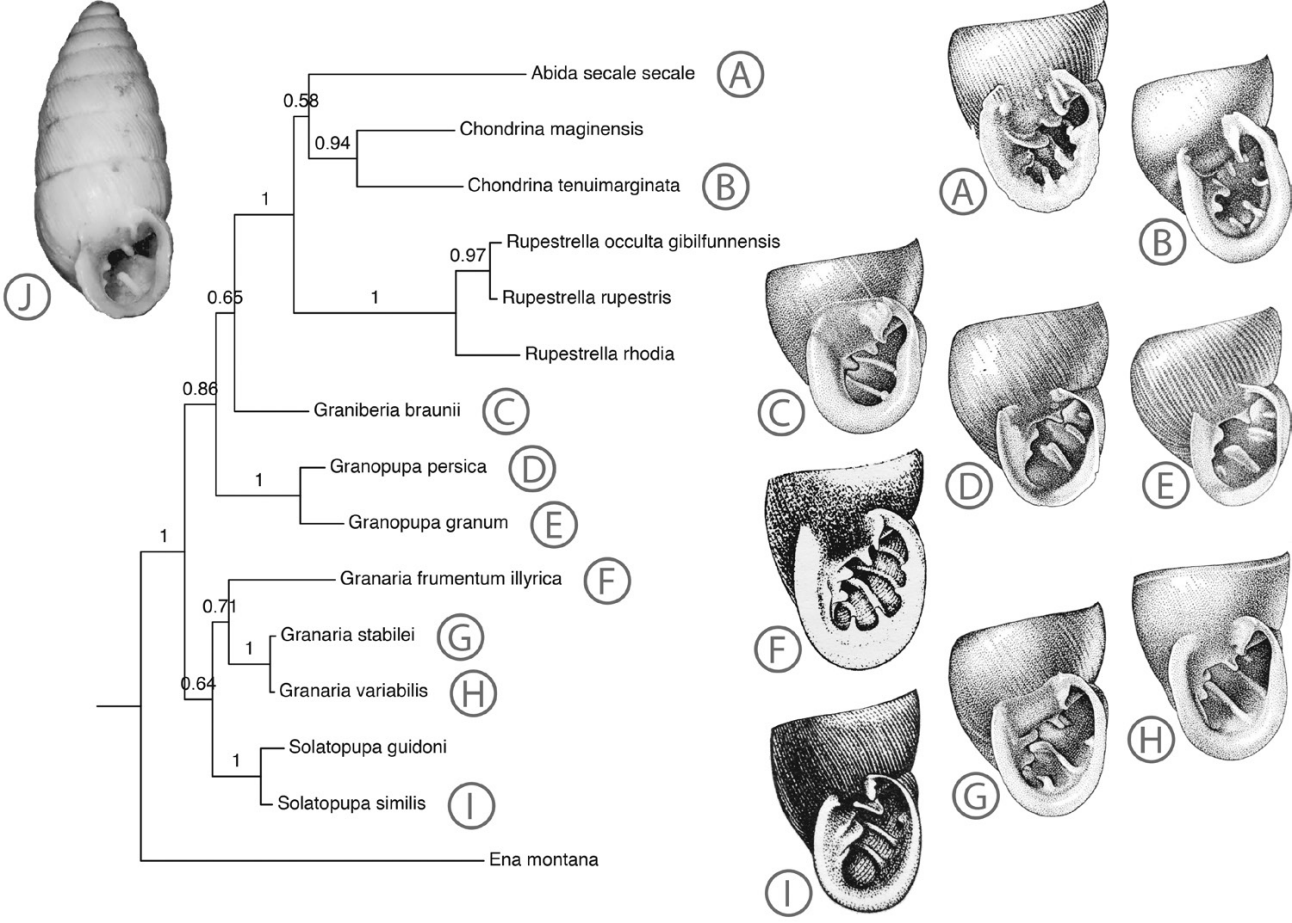
Bayesian phylogeny reconstruction of Chondrinidae based on a concatenated dataset of nucleotide (*COI* and *16S*) and amino acid (*COI*) sequences. All depicted apertures are after Gittenberger (1973), except **F** and **I** which are after Kerney, Cameron and Gittenberger (1980). Aperture **D** is not of *Granopupa persica*, but of the closely related *Granopupa arabica*. Inset J shows a photo of *Granaria grossecostata*, after Höltke and Rasser (2013: fig. 5) (with permission).

The separate molecular analyses did not result in entirely congruent results (see Supplementary Information), so that the summarizing cladogram that is presented here (Fig. 4) has uncertainties in it. The phylogenetic relationships of all the genera remains unresolved. All reconstructions indicate, however, that *‘Granaria’ persica* is most closely related to *Granopupa granum*. The position of *Granaria braunii* is unclear, but none of the reconstructions assigns that species to a clade with *Granaria frumentum* or *Granaria stabilei*.

For data regarding the European representatives of the chondrinid species, we refer to Gittenberger (1973) and Kokshoorn and Gittenberger (2008, 2010).

## Discussion

For genera and species of the extant Granariinae we refer to Kokshoorn and Gittenberger 2010. DNA sequence data from this study do not allow (and were neither intended) to test for the monophyly of Granariinae or Chondrininae.

In the *Granaria* species of which the genital tract had been studied in some detail (Gittenberger 1973), viz. the type species *Granaria frumentum* (Draparnaud, 1801), *Granaria variabilis* (Draparnaud, 1801), *Granaria stabilei* (E. von Martens, 1865), and *Granaria braunii* (Rossmässler, 1842), the male loop is provided with a prominent flagellum. In these species, the lumen of the entire proximal half of the loop, which may be considered homologous with the penis, has some longitudinal ridges only, which continue into the flagellum; before the transition into the vas deferens, the lumen is covered with many fine papillae. The *Solatopupa* species, which differ in shell shape and the structure of the radula, have the same bauplan of the genital tract (Gittenberger 1973).

According to molecular analyses (Kokshoorn and Gittenberger 2008 and Fig. 4), the W European *Granaria variabilis* and *Granaria stabilei* are sisterspecies (Fig. 4 and Suppl. material 1; pp. 1.0 and 0.86); they may have evolved from a common ancestor as lowland and alpine descendant, respectively. The polytypic Central & E European *Granaria frumentum* (see Fehér et al. 2010) could be the sistertaxon of their ancestral species (Fig. 4; pp. 0.71), but the *COI* and *16S* phylogenies (Suppl. materials 1, 2) are inconclusive. In the latter phylogeny *Granaria frumentum* is the sistertaxon of *Solatopupa* (Suppl. material 1; pp. 0.97), whereas with *COI Granaria* and *Solatopupa* are not monophyletic (though both are outside the clade Chondrininae-*Granopupa*). The summarizing cladogram indicates *Granaria* and *Solatopupa* as sistergroups (Fig. 4; pp. 0.64), but their monophyly is only supported by *16S* (Suppl. material 1; pp. 0.98).

The radula of *‘Granaria’ persica* has the bauplan that is considered plesiomorphic because it is known from *Granaria*, *Granopupa*, *Abida* and snails of many other pulmonate genera that are not feeding on algae or lichens and are not obligatory rock-scraping (Gittenberger 1973, Breure and Gittenberger 1982). The radulae of both *Chondrina* and *Rupestrella* have the apomorphic rock-scraping condition, i.e. a series of virtually identical unicuspid teeth in the central part of the rows of teeth (Breure and Gittenberger 1982).

In *‘Granaria’ persica*, there is no flagellum and, according to the luminal structure, the penis is restricted to the proximal third of the male loop; the segment of the loop before the vas deferens is devoid of small papillae. According to the structure of the genitalia, *Granaria persica* and *Granopupa granum* are sistergroups and, as a consequence, should be considered congeneric. This view is convincingly supported by the molecular phylogenies (Fig. 4, Suppl. material 1, 2; pp. 1.0, 1.0, 0.86), which also show *Granopupa granum* and *Granaria persica* as sistergroups. We suggest to classify in *Granopupa* the three chondrinid species from the eastern part of the range of the family, that were classified in *Granaria* by Gittenberger (1973), and considered closely related, viz. *Granopupa arabica* (Dohrn, 1860), *Granopupa lapidaria* (Hutton, 1849) and *Granopupa persica* (Gittenberger, 1973).

The Iberian *‘Granaria’ braunii* does belong to neither the otherwise monophyletic group *Granaria* (Fig. 4, Suppl. material 1; pp. 0.71, 0.75) nor to the clade *Granaria*-*Solatopupa* (Fig. 4, Suppl. material 2; pp. 0.64, 0.98), as defined above. Morphologically *‘Granaria’ braunii* cannot be distinguished from *Granaria* and *Solatopupa* on the basis of the structure of the genital tract, whereas the apertural armature of the shell has the *Granaria* & *Granopupa* bauplan with a most prominent palatalis inferior. Apart from that, however, *‘Granaria’ braunii* is not particularly similar to any of the other chondrinids, and therefore, short of molecular data, its closest relative was considered unknown by Gittenberger (1973: 62). The molecular phylogenetic analyses that could be performed now (Fig. 4, Suppl. material 1, 2) indicate that *‘Granaria’ braunii* should not be classified with *Granaria* (i.e. *Granaria frumentum*, *Granaria variabilis* and *Granaria stabilei*). Hence we introduce a monotypic genus for this species.

### Graniberia

Taxon classificationAnimaliaPulmonataChondrinidae

Gittenberger, Groenenberg & Kokshoorn gen. n.

http://zoobank.org/2B716941-2F7D-436A-A4B9-FA53835D5241

#### Diagnosis.

Columellaris much more prominent than the infracolumellaris, which is not or hardly visible in frontal view; palatal lamellae reaching their maximum prominence clearly deeper than half a whorl inside the last whorl; apertural lip strongly reflected and broadly thickened.

#### Type species.

*Graniberia braunii* (Rossmässler, 1842) Figure 4C.

#### Remarks.

The three extant *Granaria* species, the fossil taxa that are currently classified with *Granaria*, and the four known *Granopupa* species, all differ from *Graniberia braunii* in the characters mentioned in the diagnosis.

In all *Granaria* and *Granopupa* species and in the other Chondrinidae species with an apertural dentition that is not reduced, viz. several *Abida* and *Chondrina* species, the infracolumellaris is clearly visible. Therefore, an obsolete infracolumellaris as in *Graniberia braunii*, is considered the apomorphic character state. For both the location of the palatal lamellae and the prominence of the apertural lip this is also concluded. A similar reasoning is accepted here, with only the marginal note that very few *Abida* and *Chondrina* species have more or less clearly developed a thickened apertural border, whereas in only very few *Abida* species deep palatal folds occur.Two subspecies are currently recognized, viz. *Graniberia braunii braunii* (Rossmässler, 1842) and *Granaria braunii marcusi* (Gittenberger & Ripken, 1993).

#### Derivatio nominis.

*Graniberia* after the distribution of a genus resembling *Granaria* in the Iberian peninsula.

### Monophyly of the *Granaria* s.l. species

There are no conchological differences known to distinguish between *Granaria* and *Granopupa*. Even the disputable use of a difference in shell size in not tenable anymore. As a consequence, the generic classification of the fossil so-called *Granaria* species is problematic. Unless an overlooked diagnostic character of the shells will be discovered, it will remain impossible to decide in a particular case for either *Granopupa* or *Granaria* on the basis of shell morphology. The generic classification of the fossil chondrinids is problematic anyway, because the diversification of the chondrinid lineages may have taken place an unclear period of time after the Eocene, as is suggested by the fossil record, and by the application of a molecular clock model to the molecular phylogenetic reconstruction (Kokshoorn and Gittenberger 2008). Thus, the oldest ‘*Granaria*’ species, which is known from the Eocene, and the taxa from younger deposits [see Höltke and Rasser (2013)], may be ancestral to the species in the combined six genera that are now considered to constitute the Chondrinidae. The genus *Granaria*, as it is actually accepted in the literature for both extant and extinct species might be polyphyletic.

## Supplementary Material

XML Treatment for Graniberia

## Supplementary materials

Supplementary material 1 Supplementary figure 1 Data type: PDF file Explanation note: Bayesian phylogeny reconstruction of Chondrinidae based on *COI* (nucleotide + amino acid) sequence data. This dataset is made available under the Open Database License (http://opendatacommons.org/licenses/odbl/1.0/). The Open Database License (ODbL) is a license agreement intended to allow users to freely share, modify, and use this Dataset while maintaining this same freedom for others, provided that the original source and author(s) are credited.Edmund Gittenberger, Bas Kokshoorn, Ulrich Bößneck, Bastian T. Reijnen, Dirk S.J. Groenenberg

Supplementary material 2 Supplementary figure 2 Data type: PDF file Explanation note: Bayesian phylogeny recontruction of Chondrinidae based on *16S* sequence data. This dataset is made available under the Open Database License (http://opendatacommons.org/licenses/odbl/1.0/). The Open Database License (ODbL) is a license agreement intended to allow users to freely share, modify, and use this Dataset while maintaining this same freedom for others, provided that the original source and author(s) are credited.Edmund Gittenberger, Bas Kokshoorn, Ulrich Bößneck, Bastian T. Reijnen, Dirk S.J. Groenenberg
