# Supplementary figures and images for: Granopupa in Iran, monophyly, and the fossil Granariinae (Gastropoda, Pulmonata, Chondrinidae)
Source: Zookeys. 2016 May 25;(592):27–37. doi: 10.3897/zookeys.592.7907 (PMC4926635; doi:10.3897/zookeys.592.7907)

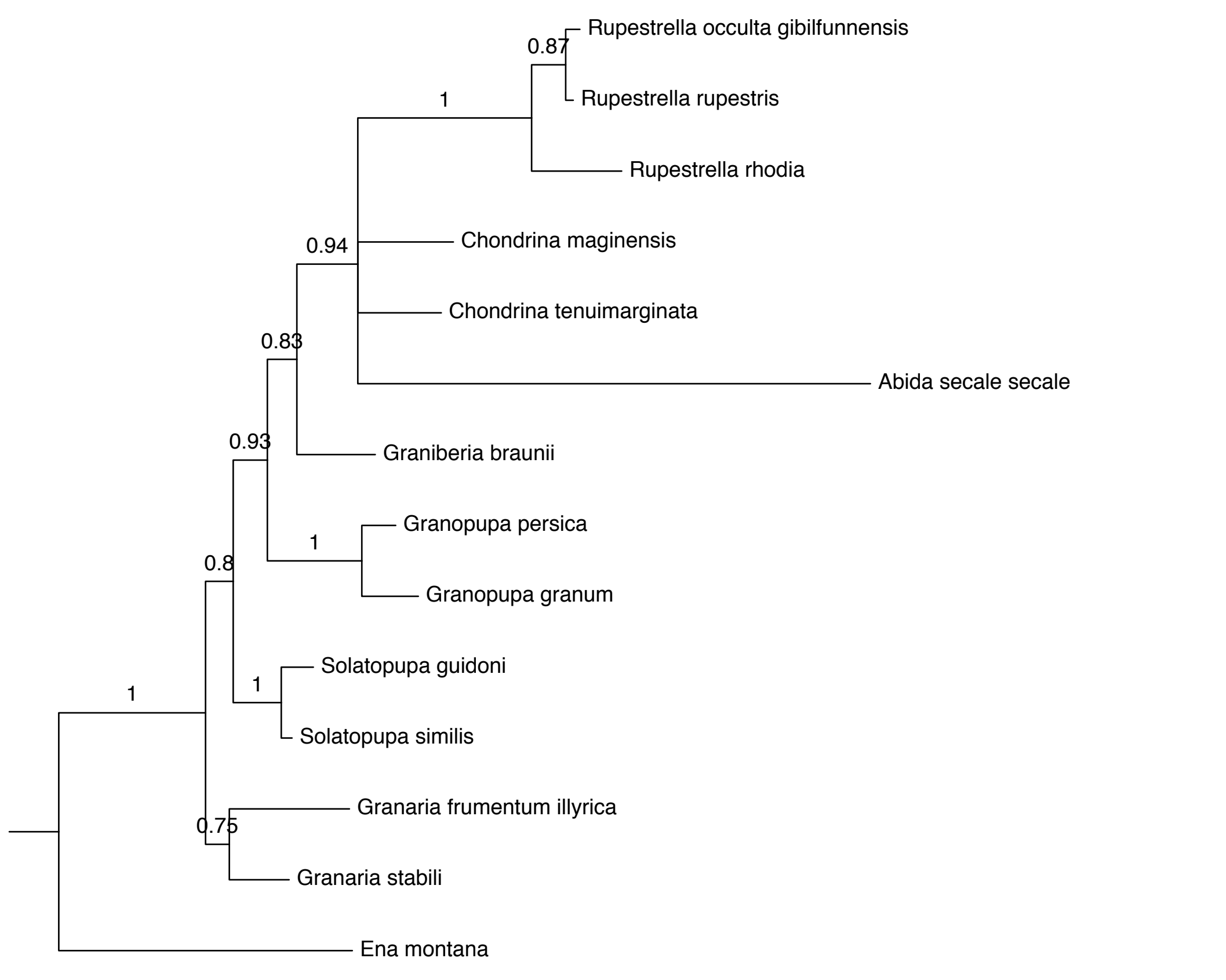

Supplement: Supplementary material 1 — Supplementary figure 1 [file zookeys-592-027-s001.pdf]

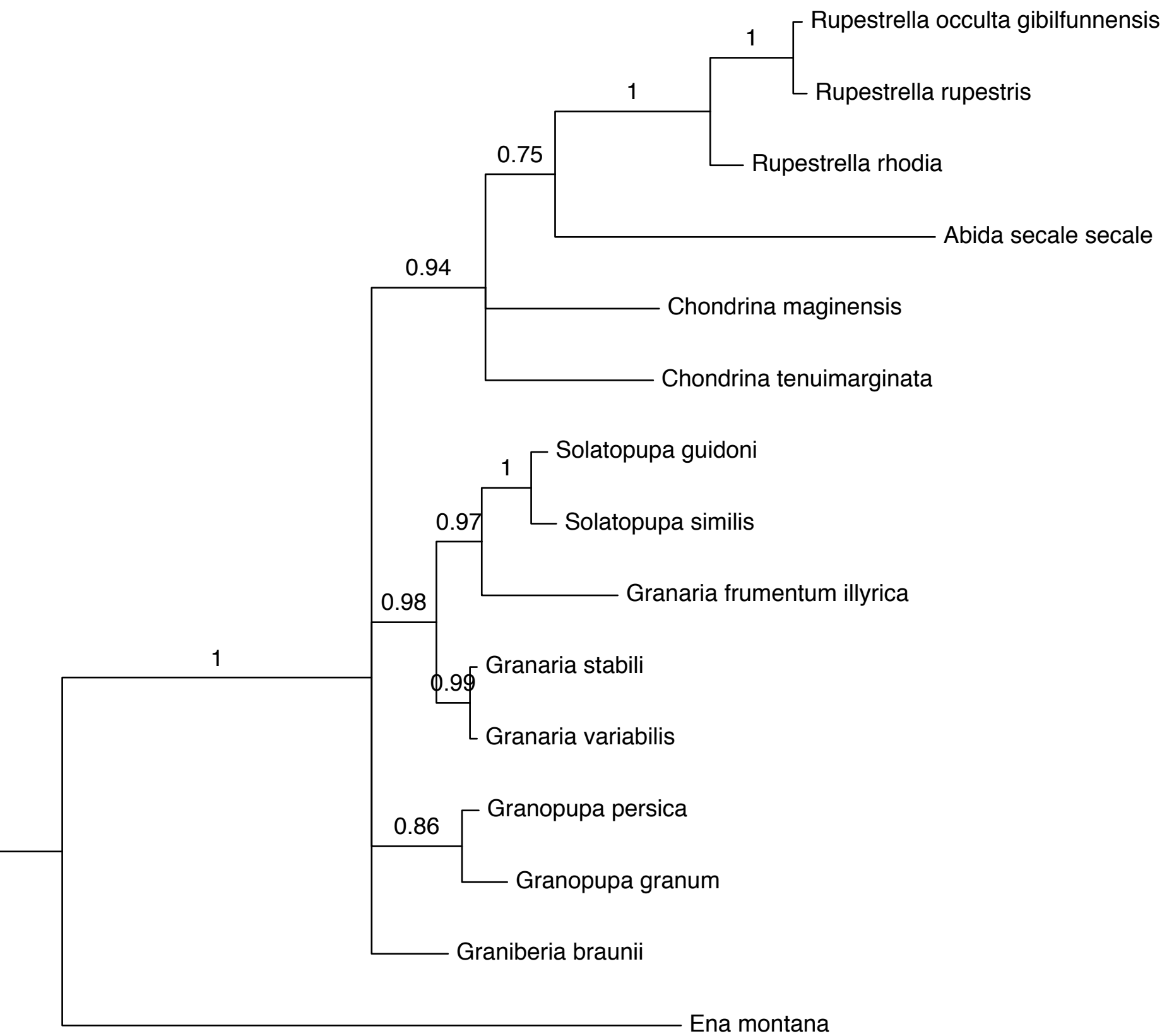

Supplement: Supplementary material 2 — Supplementary figure 2 [file zookeys-592-027-s002.pdf]
